# Effectiveness and Toxicities of Intensity-Modulated Radiotherapy for Patients with Locally Recurrent Nasopharyngeal Carcinoma

**DOI:** 10.1371/journal.pone.0073918

**Published:** 2013-09-10

**Authors:** Hai-yan Chen, Xiu-mei Ma, Ming Ye, Yan-li Hou, Hua-Ying Xie, Yong-rui Bai

**Affiliations:** Department of Radiation Oncology, Renji Hospital, School of Medicine, Shanghai Jiao Tong University, Shanghai, China; IRCCS National Cancer Institute, Italy

## Abstract

**Purpose:**

To analyze the effectiveness and toxicities in the re-irradiation of locally recurrent nasopharyngeal carcinoma (NPC) using intensity-modulated radiotherapy (IMRT).

**Methods:**

This is a retrospective analysis of 54 NPC patients with local recurrence re-irradiated with IMRT. The re-staging for rT1, rT2, rT3, rT4 were 3 (5.6%), 8 (14.8%), 9 (16.7%), 34 (63%) respectively. The average dose to GTV was 69.95 Gy (49.8-76.58 Gy), the average BED_3Gy_ was 116.8 Gy (83.5-127.9 Gy). V_95_ was 96%, and D_95_ was 65.75Gy. 33.3% of them received concurrent chemoradiotherapy.

**Results:**

Median overall survival (OS) was 21 months (1-93 mon). The 1-, 2-year local progression free survival (LPFS) rate was 84.5%, 64% and OS rate was 71.7%, 44.3%. Severe late adverse events (SLAE) occurred in 48.1% of patients, including 31.5% with ulcer or necrosis of the nasopharyngeal mucosa, 20.4% with difficulty in feeding, 18.5% with temporal lobe necrosis, 11.1% with massive hemorrhage. 15.4% died of local regional progression, 5.8% died of distant metastasis, 25% died of SLAE, 9.6% died of both local regional progression and SLAE that could not be differentiated, 5.8% died of other medical complications. Concurrent chemoradiotherapy was the independent negative prognostic factors for LPFS; PTV>100 ml was a predictive factor of poor OS; patients with invasion of post-styloid space were at higher risk of SLAE.

**Conclusions:**

The present study demonstrated that IMRT with 70Gy was efficient for local tumor control. However, we observed a high frequency of serious late complications. More optimized combination treatment and patient selection are required to achieve excellent local control without significant late morbidities in locally recurrent NPC.

## Introduction

Nasopharyngeal carcinoma (NPC) is a common malignant tumor in China. With the development of modern imaging and irradiation techniques, the 5-year overall survival (OS) of newly diagnosed NPC patients without distant metastasis has reached 75% after conventional external beam radiotherapy (EBRT) in Asian patients [1]. However, 10-36% of patients had local recurrence [2,3]. EBRT, brachytherapy and stereotactic radiosurgery have been used to re-irradiate locally recurrent disease. Each method has its optimal treatment population [4]. Early data have suggested that the use of IMRT for retreatment of local NPC recurrence is clinically feasible and could produce acceptable disease control. However, treatment-induced adverse effects from re-irradiation with IMRT are not negligible, some of which are quite severe. The aim of this report is to assess the efficacy and late toxicities of 54 patients with locally recurrent NPC treated with a uniform IMRT technique.

## Material and Methods

### 1.1

Between June 2005 and May 2012, a total of 54 patients with locally recurrent NPC were diagnosed and treated with IMRT according to our institutional treatment protocol in Renji Hospital, Shanghai Jiaotong University. This project was approved by an authorized human research review board in our institute (Ethics Committee of Renji Hospital). All the patients provide their written informed consent to participate in this study. These 54 patients were analyzed for tumor control, survival and radiation-induced adverse effects.

Local recurrence was histologically confirmed in 39 of the 54 patients (72.2%). The other 15 patients were diagnosed according to radiological examinations and clinical manifestations. All patients had MRI of head and neck, 14 of whom also had PET-CT scan. All cases were restaged according to the American Joint Cancer Committee 2010 staging classification. The numbers of patients with rT1, rT2, rT3, rT4 were 3 (5.6%), 8 (14.8%), 9 (16.7%), 34 (63%) respectively. Thirty-six patients (66.7%) had only local recurrence, and the other 18 patients (33.3%) had both local recurrence and cervical lymph node metastasis. The baseline characteristic was illustrated in Tab. 1.

**Table 1:** Baseline and treatment characteristics for 54 patients with locally recurrent NPC.

Patients characteristics					
Age	≤60	41 (75.9%)	Sex	Male	44 (81.5%)
	>60	13 (24.1%)		Female	10 (18.5%)
Initial stage	Ⅰ	3 (5.6%)	rT stage	rT1	3 (5.6%)
	ⅠⅠ	6 (11.1%)		rT2	8 (14.8%)
	ⅠⅠⅠ	33 (61.1%)		rT3	9 (16.7%)
	ⅠV	10 (18.5%)		rT4	34 (63%)
Recurrent stage	StageⅠ-ⅠⅠ	8 (14.8%)	Initial chemotherapy	No	23 (42.6%)
	StageⅢ-ⅠV	46 (85.2%)		Yes	31 (57.4%)
Fractionated dose	2 Gy/Fx	44 (81.5%)	Cervical LN	With	17 (31.5%)
	2.33 Gy/Fx	10 (18.5%)		Without	37 (68.5%)
Time interval (mon)	<24	20 (37%)	Pathology confirm	38 (70.4%)	
	24-60	16 (29.6%)	PET-CT	16 (29.6%)	
	60	18 (33.3%)	Initial RT dose	71.62Gy	

### 1.2: Distribution of recurrent lesions

Recurrent lesions were divided by anatomical structure. Skull base, prestyloid space, poststyloid space were most commonly invaded, which occupied 77.8%, 75.9%, 57.4% respectively, followed by retropharyngeal space 50%, intracranial cavity 42.6%, sphenoid sinus 40.7%, esthmoid sinus 22.2%, orbit 16.7% and so on.

### 1.3: Radiotherapy

All patients were immobilized in the supine position with thermoplastic masks. CT simulation with intravenous contrast medium using of 3-millimeter slices from the vertex to 2 centimeters below the clavicular heads was performed. The imaging data were transferred to a Pinnacle^3^ planning system. The gross tumor volume (GTV), clinical target volume (CTV), planning target volume (PTV), cervical lymph node tumor volume (GTVnd) and organs at risk (OAR) were contoured slice by slice on computed tomography images. The GTV encompassed the extent of the tumors defined by the computed tomography/ MRI imaging studies. The clinical target volumes (CTVs) of both GTV-P and GTV-N were designed to encompass microscopic disease by a margin expansion as much as 5 to 8 millimeters to both GTVs, with smaller margins (-1 mm) close to critical intracranial structures or the spinal cord. Prophylactic irradiation to high-risk areas, including draining lymphatics (i.e., uninvolved cervical lymph nodes), was not administered. An additional 3-millimeter margin was added to the CTV to create the PTV to allow for setup variability and internal motion. All patients received full-course IMRT with 6 MV X-rays generated by a Clinac-600C linear accelerator (Varian Medical Systems, Palo Alto, CA, USA) with 7 or 9 beams. 2.0 Gy/Fx was given to 44 patients (81.5%), 2.33 Gy/Fx to the other 10 patients (18.5%), one fraction per day, five day per week. The average dose to GTV was 69.95 Gy (49.8-76.58 Gy), the average BED_3Gy_ was 116.8 Gy (83.5-127.9 Gy). The percentage of GTV receiving 95% of the prescribed dose (V_95_) was 96%, and the dose encompassing 95% of the GTV (D_95_) was 65.75Gy. The range and mean doses of the OARs are listed in Tab. 2. One patient ended the treatment at 50 Gy because of pulmonary infection. Another patient received 50 Gy because of the short interval of 6 months after his first radiation treatment.

**Table 2:** IMRT doses of critical structures and dose-volume histogram irradiated with recurrent NPC.

	Average (range)		Average (range)
GTV	61cc (5-200)	left TMJ	31.19Gy (1.3-72.24)
PTV	136cc (31-435)	right TMJ	36.07Gy (1.24-71.79)
GTV average max dose	76.46Gy (52.08-84.07)	Left Optic nerve	24.61Gy (0.78-68.92)
GTV average min dose	56.88Gy (10.65-72.77)	Right Optic nerve	24.31Gy (0.45-72.01)
GTV average dose	70.95Gy (49.8-76.58)	Optic chiasm	30.17Gy (0.8-71.13)
D_95_	6575cGy (0.99-75.92)	Brain stem	24.06Gy (1.16-54.36)
V_95_ (100%)	96%(47%-100%)	Spinal cord	9.99Gy (0.29-53.76)
PTV average dose	72.7Gy (48.5-72.91)	Left len	5.41Gy (0.67-62.38)
left temporal lobe	22.64Gy (0.75-63.92)	Right len	6.06Gy (0.73-71.57)
left temporal lobe	23.07Gy (0.67-63.05)	pytuitary	4.46Gy (0.7-75.4)

Abbreviations: TMJ= Temporomandibular joint

Twenty eight patients (51.9%) received chemotherapy after their recurrence, 18 of them (33.3%) received concurrent chemoradiotherapy, 7 received neo-adjuvant chemotherapy, and 12 received adjuvant chemotherapy. The chemotherapy regimens included taxol and cisplatin in 20 patients, 5-Fu and cisplatin in six patients, gemcitabine and cisplatin in two patients. Six patients were given epidermal growth factor receptor (EGFR: cetuximab) weekly.

### 1.5: Follow-up

All patients were examined weekly during their treatment period and were required to be followed up by their attending physicians after the completion of IMRT every 3 months in the first 2 years, every 6 months for 3 additional years, and annually thereafter according to our institutional protocol of NPC management. Each follow-up visit included a complete history and examination, flexible fiberoptic nasopharyngoscopy, chest X-ray, ultrasound or CT of the abdomen, and MRI of the head-and-neck region was also required at each follow-up visit. Adverse events secondary to treatment were accessed and scored according to the radiation morbidity scoring criteria of the RTOG. Severe late adverse events (SLAE) included those late adverse events ≥Grade Ⅲ observed 3 months after the completion of re-irradiation with IMRT described by RTOG. Since some important and common late effects such as difficulty in feeding and epistaxis are not described and graded by RTOG, we graded both adverse effects according to CTCAE 3.0[5], and those ≥Grade Ⅲ were also defined as SLAE in this study.

### 1.6: Statistics

The duration of time to locoregional failure and metastasis was defined from the date of the first day of IMRT to locoregional or distant failure. The duration of OS was defined from the onset of IMRT to death or the date of the last follow-up visit. The rates of local progression-free survival (LPFS), and OS were analyzed by the Kaplan-Meier method. Log-rank tests were used to detect differences in survival between different prognosticators. Multivariate analysis with the Cox proportional hazard model was performed for all potential prognosticators. The level of significance was set at a two-tailed p value of <0.05.

## Results

### 2.1: Local control and survival

The median follow-up was 16.5 months (1-93 mon) for the entire group, and 26 months (12-93 mon) for the survivors. At the time of this analysis, five patients were censored and 22 lived of last follow-up. 21 patients (38.9%) had locoregional failure (all of them had local failure inside the 95% of the prescribed dose curve except one), six (11.1%) had distant metastasis, 35 (64.8%) were dead. Median OS was 21 months (1-93 mon). The 1-, 2-year LPFS rate was 84.5%, 64% and OS rate was 71.7% and 44.3%.

### 2.2: Toxicities

Acute adverse events: ≥Grade Ⅲ mucositis were observed in 21 patients (38.9%), and 23 patients (42.6%) had GradeⅠ or Ⅱ mucositis.

Late adverse events: The common late normal tissue effects observed before re-irradiation included Grade I xerostomia with the incidence rate of 15.2%, Grade I hearing deficit with the incidence rate of 22.2%, trismus with the incidence rate of 16.7%. After re-irradiation, 33 patients (61.1%) had Grade II trismus, 20 patients (37.4%) had Grade I or II cranial nerve palsy, two patients (3.7%) with Grade Ⅲ cranial nerve palsy; 26 patients (48.1%) had Grade I hearing deficit; 10 patients (18.6%) had Grade II hypomnesis; one patient (1.9%) had Grade I hypopsia.

**Figure 1:**
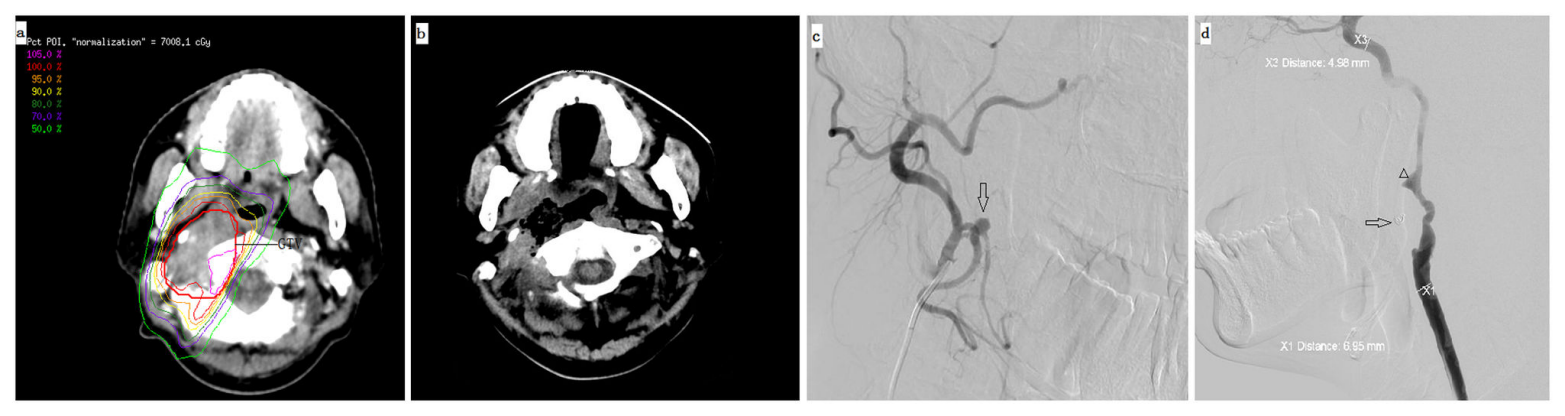
One patient who had haemorraghe after reirradiation: (a) showed the treatment plan for a patient with recurrent foci invading the poststyloid space and so on, (b) showed the CT scan four months after IMRT of the necrosis of nasopharyngeal mucose, and a cavity continuing from the pharynx was formed after the tissue was expelled, (c) the patient had several attack of haemorraghe, DSA showed the right lingual artery pseudoaneurysm (arrow) five months after IMRT, (d) the patient again had massive haemorraghe six months after IMRT, DSA showed pseudoaneurysm in right internal carotid artery (arrow head), coil (arrow) placed at the lingual artery level for (c).

No SLAE was observed before re-irradiation, after re-irradiation 26 patients (48.1%) developed SLAE, including 17 patients (31.5%) with ulcer or necrosis of the nasopharyngeal mucosa, 11 patients (20.4%) with difficulty in feeding, ten patients (18.5%) with temporal lobe necrosis (TLN), six patients (11.1%) with massive hemorrhage (Figure 1).

**Figure 2:**
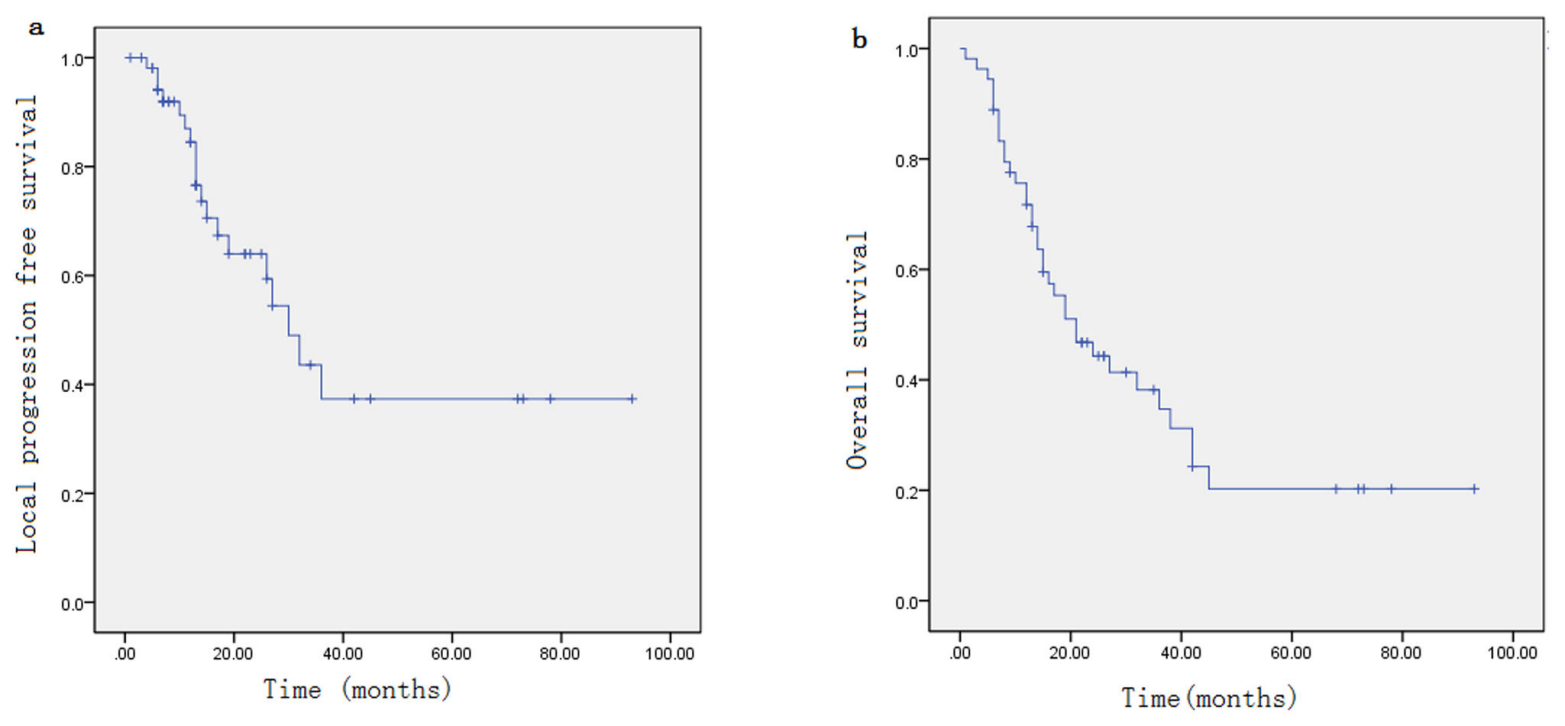
Kaplan-Meier curves showing local progression-free survival (a), overall survival (b)

### 2.3: Survival and SLAE analysis (Figure 2)

The following covariates were examined in our statistical models: age, initial TNM stage, chemotherapy at initial treatment (yes/no), the time interval between initial radiation and re-irradiation, type of recurrence (with or without cervical lymph node metastasis), rT stage, rN stage, recurrent stage, GTV, PTV, BED_3Gy_ at re-irradiation, fractionated dose, concurrent (yes/no), adjuvant chemotherapy (yes/no), and distribution of recurrent foci. Tab. 3 showed the results of univariate analyses for OS, LPFS. Tab. 4 showed the results of multivariate analyses. In the multivariate analysis, PTV>100 ml was an independent negative prognostic factor for OS (P=0.008). Concurrent chemoradiotherapy (P=0.019) was the negative prognostic factor for LPFS. Patients with concurrent chemoradiotherapy had shorter local progression free survival. Based on this result, we further analyzed the distribution of rT stage, rTNM, PTV, GTV, sex, age group, RT dose between the concurrent arm and nonconcurrent arm, and found that the distribution was equal except for the rT stage. There was more rT3-4 in the concurrent arm, with P value of 0.077.

**Table 3:** Univariate analyses of survival and local progression free survival for recurrent NPC after IMRT.

item		OS		LPFS	
		2-year	P	2-year	P
Cervical LN	With	31.1%	0.087	58%	0.211
	Without	50.7%		67.4. %	
BED_3Gy_	≤116.9	48.5%	0.642	59.4%	0.989
	>116.9	34%		65.4%	
rGTV	≤50ml	55.4%	0.031	70.3%	0.125
	>50ml	24.1%		48.9%	
rPTV	≤100ml	66.8%	0.005	82.6%	0.018
	>100ml	25.1%		43%	
CRT	no	52%	0.334	75%	0.014
	yes	29.3%		15.6%	
rT stage	rT1-2	50%	0.901	58.9%	0.953
	rT3-4	54%		65.9%	
Recurrent stage	Ⅰ-Ⅱ	57.1%	0.901	54.7%	0.998
	Ⅲ-Ⅳ	54.8%		65.6%	
Prestyloid space	no	55.9%	0.478	48.9%	0.900
	yes	41.3%		70.1%	
Poststyloid space	no	54.9%	0.022	71.2%	0.072
	yes	36.5%		59.5%	
Retropharyngeal space	no	55.8%	0.062	54.5%	0.799
	yes	32.6%		67.5%	
Massetric space	no	43.3%	0.576	71.1%	0.016
	yes	50%		20%	
Intracranial cavity	no	35.5%	0.136	62.7%	0.899
	yes	56.9%		66.4%	

Abbreviation: CRT= concurrent chemoradiotherapy

**Table 4:** Mutivariate analyses of survival and late severe adverse effects for recurrent NPC after IMRT.

		B	SE	Wald	df	Sig.	Exp(B)
OS	PTV	0.983	.368	7.138	1	0.008	2.673
LPFS	CRT	1.142	0.488	5.478	1	0.019	3.132

In the univariate analysis of risk factors for SLAE, poststyloid space invasion was the only factor that predicted high incidence, with P=0.007, details in Tab. 5. In the multivariate analysis, poststyloid space invasion was the independent predictive factors for SLAE, P=0.005; and all the other factors including rT stage, GTV, PTV, concurrent chemoradiotherapy or not and so on all showed no significant relationship with SLAE.

**Table 5:** Univariate analyses of severe late adverse effects for recurrent NPC after IMRT.

		NO. patients	Grade 3-5 toxicities	P
r T stage	r T1-2	11	4	0.505
	r T3-4	43	22	
GTV	≤ 50 ml	34	16	1.0
	>50 ml	20	10	
PTV	≤ 100 ml	21	10	0.779
	>100 ml	33	16	
Time interval	< 24 mon	20	12	0.434
	24-60 mon	16	6	
	>60 mon	18	8	
Fractional dose	2.0 Gy/Fx	44	10	0.169
	2.33 Gy/Fx	10	7	
Concurrent chemotherapy	No	36	18	0.777
	Yes	18	8	
Prestyloid space	No	13	3	0.056
	Yes	41	23	
Poststyloid space	No	23	6	0.007
	Yes	31	20	
Intracranial cavity	No	31	16	0.593
	Yes	23	10	
Nasal cavity	No	47	24	0.423
	Yes	7	2	
Oropharynx	No	48	24	0.670
	Yes	6	2	
Massetric space	No	48	23	1.0
	Yes	6	3	

### 2.4: Analysis of cause of death (Tab. 6)

Thirty two patients died at the time of analysis. Eight patients (15.4%) died of local regional progression, three patients (5.8%) died of distant metastasis, 13 patients (25%) died of SLAE, five patients (9.6%) died of both local regional progression and SLAE that could not be differentiated, the rest three patients (5.8%) died of other medical complications.

**Table 6:** Severe late adverse effects and cause of death.

Severe late adverse effects	
Necrosis of nasopharynx	16(30.8%
dysphagia	9 (17.3%)
Temporal lobe necrosis	9 (17.3%)
Massive hemorrhage	6 (11.5%)
	
**Cause of death**	
Local progression	8 (25%
Severe late adverse effects	13 (40.6%
Severe late adverse effects and local progression	5 (15.6%
Distant metastasis	4（9.4%
other	3（9.4%

Among the 13 patients who died of SLAE, five patients died of massive hemorrhage, two of TLN, three of ulcer or necrosis of the nasopharyngeal mucosa, three of severe difficulty in feeding and obstruction.

Massive hemorrhage had the highest fatality rate, five of the six patients who hemorrhaged died. Two of them died immediately after the attack of massive hemorrhage. One died of re-bleeding four months after twice endovascular treatments. One died of repeated hemorrhage after local compression. Two had surgery, one of them died of re-bleeding from the lingual artery and inferior alveolar artery; the other is still alive.

## Discussion

IMRT techniques are able to deliver optimal dose distributions to the tumor targets and adjacent sensitive organs, thereby offered the prospect of improving therapeutic ratios. The use of IMRT for retreatment of local NPC recurrence is clinically feasible, and was used in several studies [6–10], all of which yielded acceptable disease control. In the initial IMRT study for locally recurrent NPC, the local control rate was 100% at 9 months. In the study from Chua et al [8], after a median dose of 54 Gy of IMRT with or without stereotactic radiosurgery (SRS), 58% of patients had complete regression of primary tumor; one-year loco-regional progression-free, distant metastasis-free and overall survival rates were 56, 90, and 63%, respectively. And in the study from Han et al [7], with a median follow-up time of 25 months, the rates of 2-year locoregional recurrence-free survival and overall survival were 65.8 and 67.4% respectively after 70Gy of IMRT. In the recent study from Qiu et al [9] of 239 NPC patients with local recurrence who were re-irradiated with IMRT between 2001 and 2008, with a mean dose of 70.04 Gy to the GTV, the 5 year overall survival, local progression-free survival were 44.9 and 85.8% respectively. Compared to these study, the 2-year local control rate of 64% in the current study was similar, but the 2-year OS rate of 44.3% seemed inferior. It maybe due to the relatively late rT stage: rT3 was 16.7%, and rT4 ws 63% in our study. Studies from Chua [8] and Han et al [9] all indicated that rT stage was important prognostic factor for survival. In Chua’s study [8], significantly better 1-year local progression-free rate was observed in rT1–3 than rT4 tumor (100 vs. 35%). And the 5-year OS rate were 84.6, 67, 37.9, 32.3% for rT1, rT2, rT3, rT4 respectively in Lu’s study. Advanced T classification, especially T4 disease, may be a strong marker for the underlying locally aggressive biology of the original disease.

Another possible reason maybe the large GTV and PTV herein, the mean GTV and PTV was 59.4 ml and 134 ml respectively, which was much larger than previous study. And GTV was the prognostic factor for survival illustrated by Qiu et al [9], patients with GTV >38 ml had worse prognosis. Larger GTV may be indicative of more aggressive biology, more hypoxic cell in the foci, and more resistant to treatment. In our study PTV was the only adverse prognostic factor in multivariate analysis of OS, but not rT stage or GTV. PTV was not only representative of GTV, but also the direct reflection of the treatment field. And OS of local recurrent NPC depended on not only local control, but also the treatment related side events, which was influenced by treatment field and dose. Local failure and radiation injuries were the two major cause of death in our study. In the multivariate analysis of LPFS, concurrent chemoradiotherapy was the negative prognostic factor, for those treated with concurrent chemoradiotherapy, the LPFS was shorter. This was inconsistent with conventional view. Concurrent chemoradiotherapy did not cause more treatment break or cease in this study. It may be due to the larger proportion of rT3-4 in the concurrent arm, which may affect the local control.

The incidence of SLAE was 48.1% here, which was rather high, and among the patients dead, 37.1% were died because of radiation injuries. Compared to the existing data about IMRT for local recurrent NPC, the side events here was not surprising. As illustrated in Tab. 7, the lowest incidence was from Chua et al [8] with 25% of ≥gradeⅢ events; and the highest was from Qiu et al [9] with 70.3%, whereas it was 35.7% in the data from Han et al [7], and in the external radiotherapy arm from Lee et al [10] the incidence was 75% compared with 8% in the combined-modality arm.

**Table 7:** Data of IMRT for locally recurrent NPC.

	Lu et al [6]	Chua et al [8]	Lee et al [10]		Han et al [7]	Qiu et al [9]		Current study
			CMT	EBRT				
Case	49	31	13	16	239	70		54
rT stage								
rT1	8.1%	10%	69%	25%	6.7%	4.3%	5.6%	
rT2	18.4%	16%	15%	6%	18%	35.7%	14.8%	
rT3	22.4%	29%	15%	31%	29.7%	28.6%	16.7%	
rT4	51%	45%	0	38%	45.6%	34.3%	63%	
GTV	47.2ml	28.1ml	NR		37.69ml	NR	59.4ml	
PTV	NR	39.2ml	NR		NR	NR	134ml	
Dose	71.4Gy (68.7-75.4)	56.8 (50.2-64), 32.3% had SRS boost of 8.5-12.5Gy	45Gy of EBRT and 20Gy of brachy- therapy	59.4Gy (39.6-61)	69.94Gy (61.7-78.7)	70Gy (50-77.4)	68.5Gy (49.8-76.8)	
Chem	NR	68% of induction	93% of peri-radiation 79.3% of concurrent		49%	70% of concurrent	51.9%	
LC	100%-9m	61% -1 y	53%- 5y	52%- 5y	85.8% -5y	49.3% -3y	66.1%-2y	
OS	NR	63% -1 y	60%- 5y	57% -5y	44.9% -5y	51.9% -3y	44.3% -2y	
Late toxicity		25%	8%	75%	70.3%	35.7%	48.1%	
Brain necrosis	NR	7%	8%	18.8%	25.8%	NR	18.5%	
Soft tissue necrosis	NR	0	0	0	40.6%	15.7%	31.5%	
Cranial neuropathy	NR	10%	0	12.5%	NR	24.3%	3.7%	
Massive haemorraghe	NR	0	0	0	NR	NR	13%	
Difficulty in feeding	NR	0	0	0	NR	NR	20.4%	

In different data, the calculation of SLAE may be different, this may partly lead to the different incidence of events. Considering that some unique side effects were observed after reirradiation of NPC, such as massive haemorraghe and difficulty in feeding because of various reasons, which were common and sometimes vital in the survival outcome, but were not described and graded in the RTOG side effect grading system, we graded them by CTCAE 3.0, and those ≥gradeⅢ events were also defined as SLAE. This maybe the major difference from other data, and may partly explained the high incidence of adverse events in this study.

The major SLAE was ulcer or necrosis of nasopharyngeal mucose with as high as 31.5% of incidence. This was also the major event in the study from Qiu et al [9], where the incidence was 40.6%; and it was 15.7% in Han et al’s treatment group [7]. As Marx reported [11] irradiation may cause hypoxia, hypovascularity, and hypocellularity and may impair normal collagen synthesis and cell production, which may lead to tissue breakdown and a chronic nonhealing wound. The occurrence of nasopharyngeal necrosis is also related to personal tolerance and radiation methods, especially related to irradiation dose and course. We analyzed the corresponding treatment plans, and found that most of those necrosis mucose were located in the high dose area. And this may be part of the truth of the high incidence of necrosis after second cycle of RT. Ulcer or necrosis of nasopharyngeal mucose was a tough event, even after aggressive treatment combined of internal medicine and surgical approaches such as debridement or sequestrectomy, the consequence was still dissatisfied. The condition was aggravated with subsequent internal carotid artery erosion and difficulty in feeding that leading to exhaustion, which was also the situation in the study from Hua et al [12] and Wu et al [13].

Massive haemorraghe from internal carotid artery was another lethal side effects herein. Seven (13%) patients had massive haemorraghe, and five patients died of it, the seven patients were all combined with nasopharyngeal necrosis. In the data from Han et al [7], six patients were also died of massive epistaxis, though the details were unclear, it was hard to exclude the possibility of the epistaxis due to treatment. Though the exact incidence was not reported in the data from Qiu et al [9], they also concluded that it was important to reduce incidence of nasopharyngeal mucosal necrosis and massive hemorrhage. Second cycle of RT may lead to unrepairable injury to vessels. The subintima fibrous-like substance deposition, the degeneration and decreased elasticity of the media muscle fiber, fibrosis of adventitia, and the foam cells aggregation in vessel walls are all contributing factors to the fragility of vessels after radiotherapies [14]. Vessel dysplasia may be found after radiotherapy with a high probability, which is characterized by thinning wall and deranged structure, making them be at the risk of bleeding as pressure changes or inflammatory injuries occur. Massive haemorraghe often was sudden and unpredictable, thus often lethal, and often the patient cannot wait for treatment before they die.

Another common event was difficulty in feeding, which required nutritional support either by nasal feeding, gastrostomy feeding or total parenteral alimentation. The difficulty in feeding may be caused by trismus, swallowing pain, or nasopharyngeal necrosis, and in patients after second cycle of RT these causes were often confused and hard to be differentiated. 11 patients in this treatment group finally had difficulty in oral feeding, often the normal condition became poor and exhausted, three of them died of cachexia eventually.

The common side effects reported in other reports was TLN, which was the third most severe late events in our study, with the incidence of 18.5%. It was as high as 25.8% in the study from Han et al [7], and 8% in Chua’s study [9]. Patients with TLN usually present with mild symptoms of dizziness, forgetfulness, or temporal lobe epilepsy. Severe and even fatal complications of TLN, including convulsion, intracranial hemorrhage, mass effect causing herniation, and death, had occasionally been seen. In our group, three patients finally died of TLN. The cumulation of radical dose to the lobe from two cycle of RT, especially in those with intracranial invasion may partly lead to this side effect.

SLAE was not only common but sometimes also lethal in this study, 25% of patients finally died of SLAE. In the study from Han et al [7], as much as 34.7% of patients died of treatment related toxicities eventually. Considering the important influence of SLAE on life quality and survival, it was necessary to tell who would benefit from re-IMRT and who may suffer from irrepairable damage. And in view of that some adverse effects were quite distribution-dependent, we also analyzed the relationship between the location of recurrent foci and severe events. Invasion of poststyloid space was predictive of severe events in multivariate analysis, but not GTV or rT stage, which was different from that of Qiu et al [9]. It was reasonable because the major side effects was nasopharyngeal mucosal necrosis and subsequent haemorraghe and difficulty in feeding in our study. Nasopharyngeal mucosal necrosis was most often seen in the lateral wall of nasopharynx especially after high dose of RT. Because this was the first time to analyze the relationship between side events and location of recurrent foci, it was hard to draw conclusion if this was a common phenomenon. But based on this result, it was important to consider the recurrent foci before giving the treatment in order to protect from severe events. And patient with invasion of poststyloid space was prone to have nasopharyngeal mucosal necrosis, it was important for early follow-up and intervention of suspicious clue of side events, and more importantly, to consider what was the more proper treatment for such kind of patient.

In order to decrease the incidence of severe side events, one may lower the total radiation dose, shrink the radiation field, or combine several treatment modalities. As we know, the re-irradiation dose is an important factor for the local control of recurrent NPC. Wang [15] reported the dose–response relationship of re-irradiation to outcomes in the conventional radiotherapy era. The 5 year survival rate was 45% when more than 60 Gy was delivered; however, no patients survived when the dose was less than 60 Gy. Leo et al [16], Chang et al [17] and Leung et al [18] all proposed that RT dose of more than 60 Gy was essential for local control. It seems unreasonable to lower the radiation dose to be less than 60Gy. The dose herein was 68.5Gy, which was similar to the data illustrated in Tab. 7, but even after such high dose, local failure remained the main cause of death, this may partly be explained by the major component of rT3-4 and large tumor in our series, but it also demands some thinking about the optimal dose for disease control. If high dose is necessary, for those patients with predictable high incidence of severe treatment-related events, what is the optimal treatment options?

In the data from Lee et al [10], the combination of intracavitary brachytherapy with external beam radiotherapy compared with external beam RT alone, significantly decreased the incidence of severe side events, which was 8% and 75% respectively, without influencing the local control and overall survival. Maybe for some superficial lesions such as those invading the parapharyngeal space it was a reasonable choice. And also in the data from Chua et al [8], the median dose was 56.8Gy, and radiosurgery was offered to these patients to selectively boost the PTV that received a relatively low dose (40–45 Gy) by IMRT, with a single dose which ranged from 8.5 to 12.5 Gy prescribed to 80% isodose surface. 68% had induction chemotherapy. Their results suggest that a dose between 50 and 60 Gy could yield good control for rT1–3 but not for rT4 NPC, the side effects were less compared to other existing data in their study. Gullu et al [19] illustrated that compared with conformal radiotherapy with or without brachytherapy, robotic stereotactic radiotherapy resulted in lower incidence of serious late toxicities, that was 21% of patients in the SBRT arm and 48% in the CRT arm, fatal complications occurred in three patients (12.5%) of the SBRT arm, and four patients (14.8%) of the CRT arm. Stereotactic body radiotherapy seemed particularly useful when the tumor was large or abutting/encasing critical structure. This maybe another option for some patient, for example, those with tumors invading the intracranial structures.

Though chemotherapy showed no influence on overall survival in the current and previous studies, and the concurrent arm even had shorter LPFS herein, accordingly to the experience from the treatment of primary NPC, one cannot simply exclude the efficience of chemotherapy on the treatment of local recurrent NPC, especially for those with large tumor, but what is the optimal drug and schedule is under discussion. It may be necessary to have a prospective analysis upon the efficience of chemotherapy on recurrent NPC.

In conclusion, the present study demonstrates that IMRT with 70Gy was efficient for local tumor control. However, we observed a high frequency of serious late complications, and some of the complications were lethal. More optimized combination treatment and patient selection are required to achieve excellent local control without significant late morbidities in locally recurrent NPC.
